# WNT gene polymorphisms and predisposition to apical periodontitis

**DOI:** 10.1038/s41598-019-55293-6

**Published:** 2019-12-12

**Authors:** Letícia Chaves de Souza, Franco Cavalla, Lorena Maili, Gustavo P. Garlet, Alexandre R. Vieira, Renato M. Silva, Ariadne Letra

**Affiliations:** 1 Department of Endodontics, University of Texas Health Science Center School of Dentistry, Houston, 77054 USA; 2 Center for Craniofacial Research, University of Texas Health Science Center School of Dentistry, Houston, 77054 USA; 3 Department of Conservative Dentistry, School of Dentistry, University of Chile, Santiago, 7520355 Chile; 4 Department of Biological Sciences, Bauru School of Dentistry, University of São Paulo, Bauru, 17012 Brazil; 5 Department of Pediatrics, University of Texas Health Science Center at Houston McGovern Medical School, Houston, 77030 USA; 6 Pediatric Research Center, University of Texas Health Science Center at Houston McGovern Medical School, Houston, 77030 USA; 7 Department of Oral Biology, University of Pittsburgh, Pittsburgh, 15229 USA; 8 Department of Diagnostic and Biomedical Sciences, University of Texas Health Science Center School of Dentistry, Houston, 77054 USA

**Keywords:** Genetic predisposition to disease, Gene expression

## Abstract

Single nucleotide polymorphisms (SNPs) in WNT genes may impact gene/protein function and contribute to individual predisposition to apical periodontitis (AP). Here, we investigated the association of SNPs in/nearby *WNT3*, *WNT3A*, *WNT5A*, *WNT8A*, *WNT9B* and *WNT11* genes with AP using a case-control dataset. Cases were defined as individuals with deep caries and AP (n = 188); controls had deep caries and no AP (n = 230). Genotyping was performed using Taqman chemistry in real time PCR. Data analyses was performed using Fisher Exact tests assuming a Bonferroni correction threshold value of 0.005. Single-SNP association analysis revealed a trend for association with *WNT3* rs9890413 genotypes (*P* = 0.009) under a dominant model and allelic association for *WNT3A* rs1745420 (*P* = 0.009). Haplotypes involving *WNT3-WNT9B-WNT3A* alleles were also significantly associated with AP (*P* ≤ 0.003). Luciferase reporter assays showed higher transcriptional activity (1.4-fold) with the alternate G allele in rs1745420. Expression of *WNT3*, *WNT3A* and *WNT5A* in AP tissues was significantly higher than in control tissues, and inversely correlated with the expression of *SERPINB1*, *COL1A1* and *TIMP1* (P < 0.05). Our results suggest that WNT genes have a role in modulating AP and polymorphisms in these genes may increase susceptibility to AP.

## Introduction

Apical periodontitis (AP) represents a local immune response to the progression of microorganisms from an infected root canal space to the periapical area that results in bone resorption characterized by a periapical radiolucency^1^.

AP has heterogeneous etiology, where different bacterial combinations can result in similar clinical outcomes^2^. Furthermore, the endodontic microbiota varies between individuals and each individual presents a unique bacterial profile, regarding the type of species present and their abundance^2^. Endodontic pathogens present in teeth diagnosed with pulp necrosis and AP have been associated with the expression of mediators related to soft tissue and bone destruction in AP, such as matrix metalloproteinases (MMPs)-2 and -9, receptor activator of NFκB (RANK), RANK ligand (RANKL) and osteoprotegerin (OPG). These findings suggest a dynamic process between the microbial aggression and host responses during AP development^3^. In accordance with these previous studies, single nucleotide polymorphisms (SNPs) in disease-relevant genes, mostly belonging to immune-response related pathways, have recently been reported in association with AP predisposition^4–6^.

The Wnt/β-catenin signaling pathway, also known as the canonical Wnt pathway, controls cell differentiation, growth, proliferation, survival, and immune cell function and has been linked to several skeletal diseases and cancers^7^. Wnt interactions with Frizzled (Fzd) and low-density lipoprotein receptor related protein 5/6 (LRP5/6) receptors result in cytoplasmic accumulation of β-catenin and its translocation into the nucleus, where it binds to T-cell factor/lymphoid enhancer factor (TCF/LEF) to regulate the transcription of target genes^7^. In bone, activation of Wnt/β-catenin signaling increases bone mass by regulating stem cell renewal, stimulating pre-osteoblast replication and osteoblastogenesis as well as inhibiting osteoblast apoptosis^8^. Previous studies using animal models of AP reported a significant increase in the expression of Wnt family member 5 A (WNT5A) in AP tissues which correlated with the severity of inflammation^9^. Furthermore, additional studies showed the upregulation of WNT gene expression during periapical lesion development, and suggested that the AP-induced inflammation inhibits osteoblast differentiation via modulation of Wnt/β-catenin signaling pathway^8,10,11^.

In this study, we investigated whether SNPs in WNT genes may contribute to AP risk. We also assessed the function of associated SNPs and mRNA expression analysis of WNT genes in AP tissues.

## Results

### Genetic association analysis

We used a case-control candidate gene approach to investigate the association of SNPs in/nearby WNT genes with AP (Table 1). Our study population consisted of 188 cases with AP (individuals with deep caries and AP > 3 mm in diameter) and 230 controls (individuals with deep caries but no AP). Individuals were recruited at the UTHealth School of Dentistry at Houston and at the University of Pittsburgh School of Dental Medicine Dental Registry and DNA Repository under IRB-approved protocols and informed consent. Diagnosis of AP was obtained by an endodontist based on clinical and radiographic examinations of each individual. Genomic DNA samples were used as templates for genotyping.

**Table 1:** Details of WNT genes investigated.

Chr.	Gene	SNP ID	Base Position^a^	SNP location^a^	Alleles^a,b^
1	*WNT3A*	rs708111	228003664	Upstream	**G**/A
		rs3094912	228022114	Intron 1	**T**/C/A
		rs752107	228059650	3′ prime UTR	T/**C**
		rs1745420	228064031	Downstream	G/A/**C**
3	*WNT5A*	rs566926	55486750	Intron 6	T/A/**G**
5	*WNT8A*	rs2040862	138084300	Intron 3	**C**/T
11	*WNT11*	rs1533767	76194756	Exon 6	**G**/A
17	*WNT3*	rs9890413	46824083	Upstream	G/**A**
		rs199498	46788237	Intron 1	T/**C**/G
		rs111769	46794621	Intron 1	**C**/T
17	*WNT9B*	rs2165846	46864000	Intron 2	**A**/G

Chr., chromosome; SNP, single nucleotide polymorphism.

^a^According to Ensembl GRCh38.p12 assembly (June 21, 2019).

^b^Ancestral allele in bold reported on the forward strand.

Eleven SNPs in/nearby *WNT3*, *WNT3A*, *WNT5A*, *WNT8A*, *WNT9B* and *WNT11* genes were selected for genotyping based on published reports, locations within their respective genes, potential functional consequences (i.e., located in regulatory regions such as promoter, 3′ UTR, exon, or exon/intron boundaries), or if considered tag-SNPs as surrogates for the linkage disequilibrium blocks surrounding the contributor gene^12^ (Table 1).

Association analysis was performed in the combined datasets, and also stratified by recruitment site, considering a Bonferroni correction alpha value of 0.005. A first-pass analysis showed that two SNPs (rs566926 and rs2040862) were in deviation from Hardy-Weinberg equilibrium (HWE) which were excluded from further analysis.

Under a strict Bonferroni correction criteria (α = 0.005), analysis of the combined dataset showed a trend for positive association for *WNT3* (rs9890413) in individuals with deep caries and AP, both under allelic and dominant genotype models (P = 0.007). This association appeared to be driven by the association values obtained for the Pittsburgh dataset (P = 0.009). Suggestive association was also found between *WNT3A* rs1745420 alleles and a deep caries with AP phenotype in the Houston dataset (P = 0.009). Additional nominal associations (P < 0.05) were found for other WNT genes (Table 2 and Supplementary Tables S1–S3).

**Table 2:** Summary of single SNPs association results.

Gene	SNP Id^a^	Studied population	MAF CEU^a,b^	MAF (cases)	MAF (control)	Test	Alleles	Frequency (cases)	Frequency (controls)	P value^c^
*WNT3*	rs9890413	Pittsburgh				Genotypic	GG/GA/AA	14/49/35	12/51/71	0.03
			0.36(G)	0.39	0.28	Allelic	G/A	77/119	75/193	0.01
						Dominant	GG + GA/AA	63/35	63/71	*0.009*
						Recessive	GG/GA + AA	14/84	12/122	0.20
		Combined (Pittsburgh + Houston)				Genotypic	GG/GA/AA	22/85/70	17/80/111	0.02
			0.36(G)	0.36	0.27	Allelic	G/A	129/225	114/302	*0.007*
						Dominant	GG + GA/AA	107/70	97/111	*0.007*
						Recessive	GG/GA + AA	22/155	17/191	0.17
*WNT3A*	rs1745420	Houston				Genotypic	CC/CG/GG	10/26/42	16/33/25	0.04
			0.22(C)	0.29	0.44	Allelic	C/G	46/110	65/83	*0.009*
						Dominant	CC + CG/GG	36/42	49/25	0.01
						Recessive	CC/CG + GG	10/68	16/58	0.15

^a^According to National Center for Biotechnology GRCh38.p12 assembly (June 21, 2019).

^b^Minor allele frequency (MAF) in CEU (Caucasian European) population.

^c^Fisher exact test, Bonferroni correction, significant if α ≤ 0.005. Italic font means 0.006 ≤ P ≤ 0.009.

Significant haplotype associations were also observed and included the individually associated SNPs (Table 3 and Supplementary Table S4). In the combined dataset, the strongest association was observed for the combination of *WNT3* rs111769 and rs9890413 alleles (CG haplotype, P = 0.0002), followed by *WNT3* rs111769/rs9890413 and *WNT9B* rs2165846 alleles (CGG haplotype, P = 0.0003), *WNT3* rs199498/rs111769/rs9890413 alleles (TCG haplotype, P = 0.002), *WNT3* rs199498/rs111769/rs9890413 and *WNT9B* rs2165846 alleles (TCGG haplotype, P = 0.002), and *WNT3* rs9890413/*WNT9B* rs2165846 alleles (GG haplotype, P = 0.004) (Table 3 and Supplementary Table S4). In the Pittsburgh dataset, the strongest association was observed for the combination of *WNT3* rs111769/rs9890413 alleles (CG haplotype, P = 0.00008), followed by *WNT3* rs111769/rs9890413 and *WNT9B* rs2165846 alleles (CGG haplotype, P = 0.0002), *WNT3* rs199498/rs111769/rs9890413 alleles (TCG haplotype, P = 0.0005), and *WNT3* rs199498/rs111769/rs9890413 and *WNT9B* rs2165846 (TCGG haplotype, P = 0.0009) (Table 3 and Supplementary Table S4). In the Houston dataset, association was observed for the combination of *WNT3A* rs752107 and rs1745420 alleles (CC haplotype, P = 0.003) (Table 3 and Supplementary Table S4).

**Table 3:** Summary of haplotype analysis results.

Genes	SNPs	Dataset	Haplotype	Frequency of cases	Frequency of controls	P value^a^
*WNT3*	rs111769/rs9890413	Pittsburgh	CG	0.24	0.10	**0.00008**
		Combined		0.21	0.11	**0.0002**
	rs199498/rs111769//rs9890413	Pittsburgh	TCG	0.21	0.10	**0.0005**
		Combined		0.18	0.11	**0.002**
*WNT3*//*WNT9B*	rs9890413/rs2165846	Houston	AA	0.21	0.35	*0.007*
		Combined	GG	0.21	0.13	**0.004**
*WNT3*/*WNT9B*	rs111769/rs9890413/rs2165846	Pittsburgh	CGG	0.20	0.09	**0.0002**
		Combined		0.17	0.08	**0.0003**
		Houston	CAA	0.13	0.25	*0.007*
*WNT3*/*WNT9B*	rs199498/rs111769/rs9890413/rs2165846	Pittsburgh	TCGG	0.20	0.09	**0.0009**
		Combined		0.15	0.08	**0.002**
		Houston	TCAA	0.12	0.24	*0.009*
*WNT3A*	rs752107/rs1745420	Houston	CC	0.23	0.39	**0.003**

^a^Bonferroni correction, significant if α ≤ 0.005 (in bold). Italic font means 0.006 ≤ P ≤ 0.009.

### Functional analysis of associated SNPs

**Figure 1:**
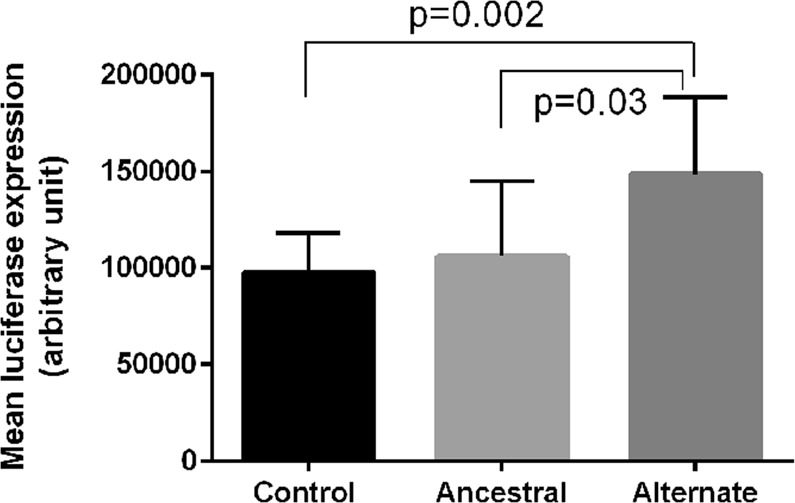
Results of luciferase reporter gene assays for *WNT3A* rs1745420. Allele-specific transcriptional activities are shown for ancestral (ANC) and (ALT) alleles.

We used the FuncPred tool on the SNPinfo web server (https://snpinfo.niehs.nih.gov/snpinfo/snpfunc.html) and TargetScan (http://www.targetscan.org) for *in silico* prediction of SNV function for the associated *WNT3* rs9890413 and *WNT3A* rs1745420. These SNVs are located upstream and downstream of their respective genes and might have potential regulatory effects on gene and/or encoded protein functions. No transcription factor binding sites were identified for *WNT3* rs9890413. For *WNT3A* rs1745420, results predicted that it may harbor eight conserved miRNA binding sites, suggesting potential effects on gene transcriptional activities (Supplementary Fig. S1). To further test for allele-specific differential transcriptional activities of *WNT3A in vitro*, a 3′ UTR reporter vector was used for plasmid construction and cloning of *WNT3A* rs174520 C and G alleles downstream of the luciferase reporter gene. Empty 3′ UTR vector was used as transfection control. Human embryonic kidney cells (HEK293T) cells were cultured following established protocols and transfected with each allele-specific construct. The results showed that the alternate allele G resulted in increased transcriptional activity (1.4-fold, P = 0.03) when compared to the ancestral allele C (Fig. 1).

### Gene expression

**Figure 2:**
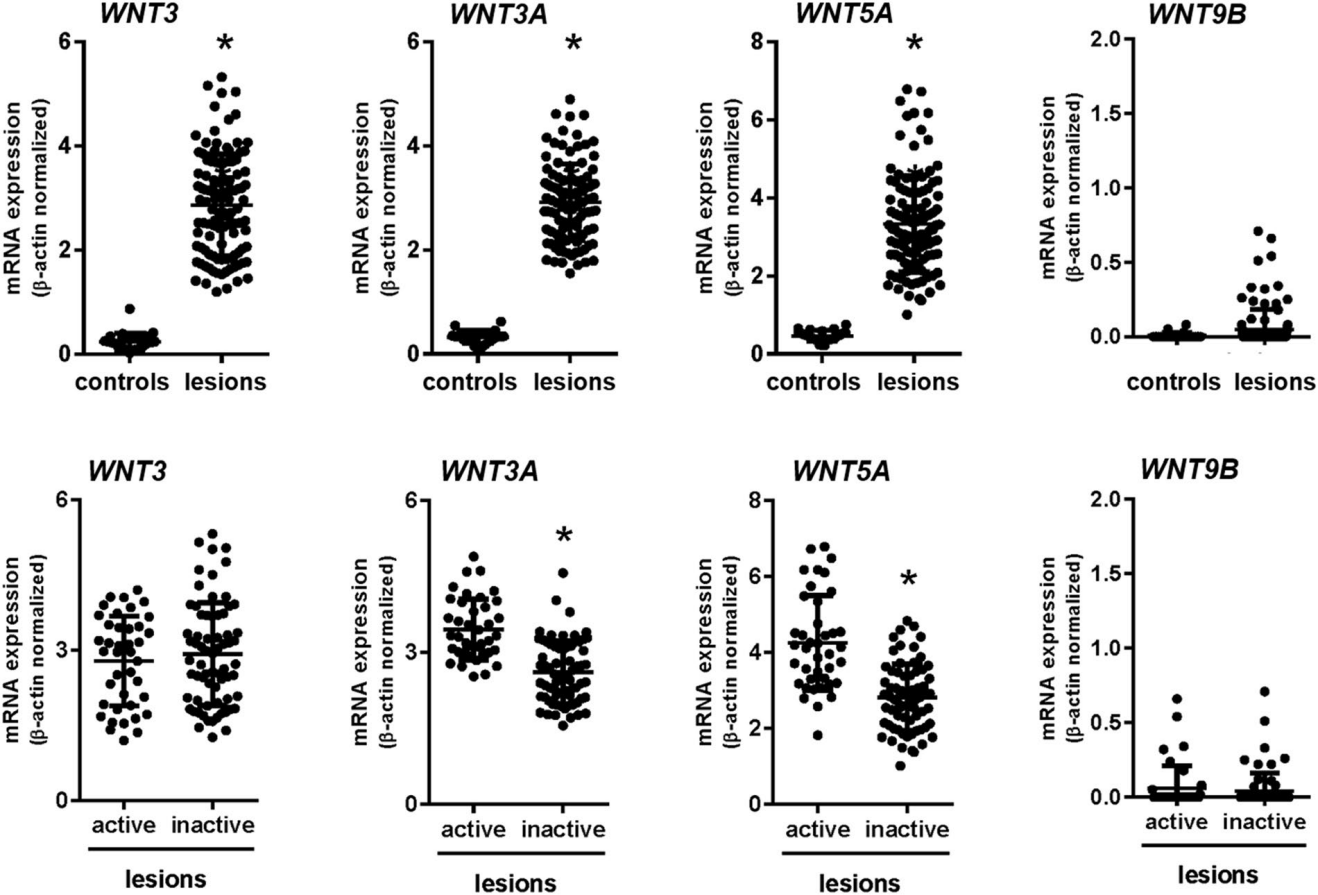
Expression of *WNT3*, *WNT3A*, *WNT5A* and *WNT9B* mRNA in apical periodontitis (AP) and control tissues. Top, mRNA expression levels in AP lesions and controls. Bottom, mRNA expression levels in active and inactive AP lesions. *Indicates P ≤ 0.05.

We investigated the mRNA expression of the associated genes *WNT3*, *WNT3A*, *WNT5A*, *WNT9B* in AP lesion tissues using quantitative RT-PCR. Healthy periodontal ligament tissues were used as controls. The expression of *WNT3*, *WNT3A* and *WNT5A* mRNA was significantly higher in AP lesions when compared to controls, whereas no differences were observed for *WNT9B* expression. We also stratified AP lesions into active and inactive status, according to the methodology proposed by Menezes *et al*.^13^, and observed that expression of *WNT3A* and *WNT5A* was markedly higher in active lesions, while no differences were observed for *WNT3* and *WNT9B* (P > 0.05) (Fig. 2).

**Figure 3:**
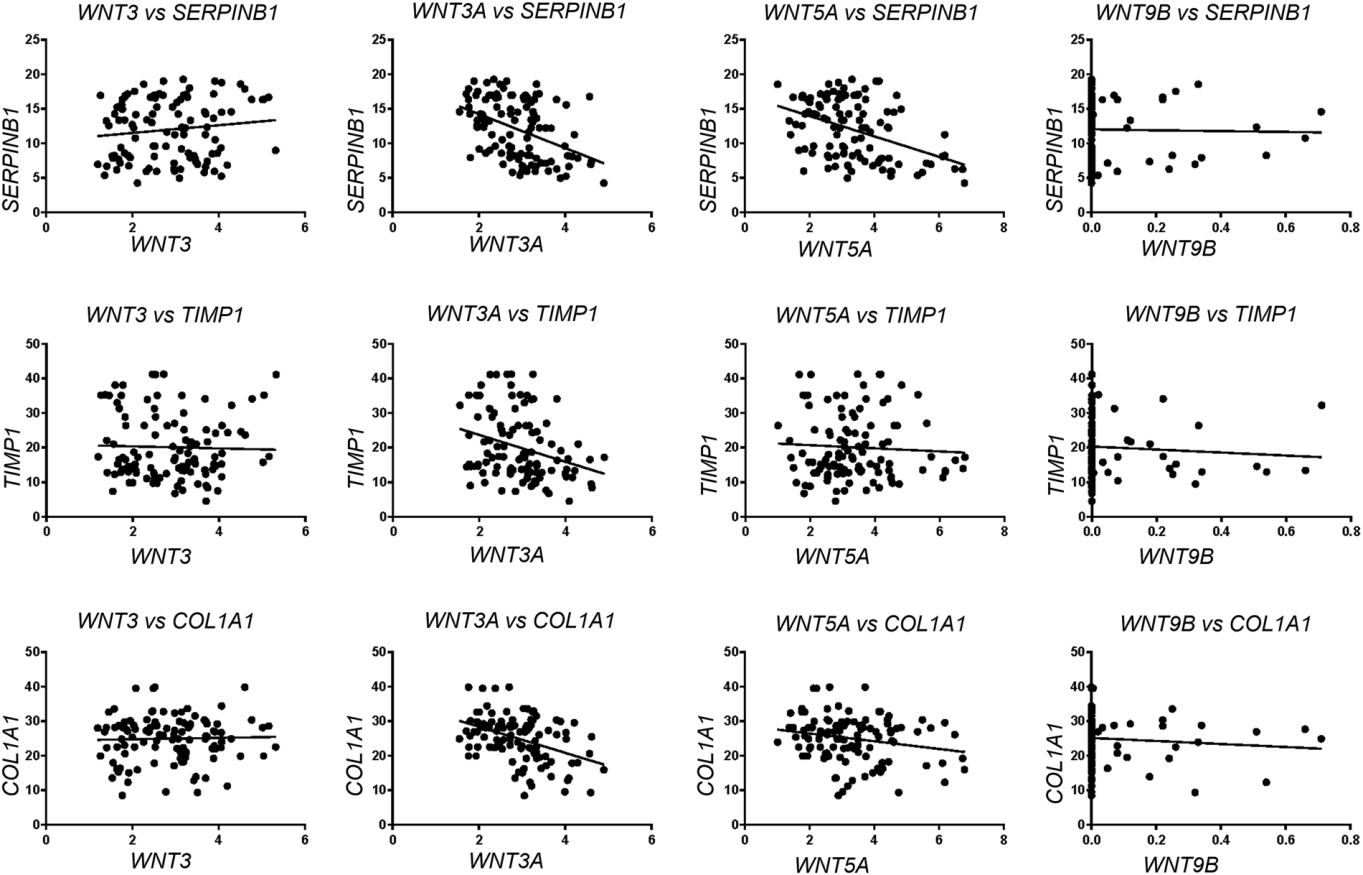
Correlation analysis between expression of *WNT3*, *WNT3A*, *WNT5A* and *WNT9B* with *SERPINB1*, *TIMP1* and *COL1A1* in AP lesions. *Indicates P ≤ 0.05.

We also performed correlation analysis to assess the potential relationships between WNT gene expression levels with the expression of genes involved in bone healing (serpin family B member 1 (*SERPINB1)*, TIMP metallopeptidase inhibitor 1 (*TIMP1*) and collagen type I alpha 1 chain (*COL1A1*). Expression of *WNT3A* and *WNT5A* was inversely correlated with the expression of *SERPINB1* (*r*^2^ = 0.1759, P < 0.0001 and *r*^2^ = 0.1798, P < 0.0001, respectively) and *COL1A1* (*r*^2^ = 0.1774, P < 0.0001 and *r*^2^ = 0.0439, P = 0.0287, respectively). Similarly, *WNT3* expression was inversely correlated with *TIMP1* expression (*r*^2^ = 0.0896, P = 0.0016). No significant correlation was observed for *WNT9B* (Fig. 3).

## Discussion

In this study, we assessed the potential association of WNT gene polymorphisms with AP risk in individuals with deep caries. We used a candidate gene association approach to investigate the association of SNPs in 11 WNT genes in 188 cases with AP and 230 control individuals without AP. Under the strict criteria of the Bonferroni correction (α = 0.005), our findings revealed a trend for association between *WNT3* (rs9890413) and *WNT3A* (rs1745420) with AP in the studied datasets.

AP has heterogeneous etiology and results from the dynamic interactions between pathogens and host defenses in the periapical tissues (i.e. those tissues surrounding the apex of a tooth), thus triggering a cascade of events with release of inflammatory mediators, local inflammation, and hard tissue breakdown characterizing an AP lesion^2^. Despite its widely accepted microbial origin, the development of AP has also been recently linked to the host immune response^4,6^. Clinical observations in individuals with deep caries vary; while in some individuals an AP lesion is detected quite readily in response to the microbial aggression, others are more resistant to developing an AP lesion in face of similar aggression. These findings are intriguing and suggest that genetic predisposition plays a role in the development of AP^6^.

Genetic polymorphisms have been reported to act as modifiers of diverse diseases and, as such, might influence the individual host response to the severity of the microbial challenge and/or the individual response to treatment of AP^14^. To date, a few studies in distinct populations and a recent meta-analysis support genetic predisposition as a risk factor towards increased AP susceptibility, with the most significant associations reported for polymorphisms in interleukin 1 beta (*IL1B*), heat shock 70 kDa protein 1-like (*HSPA1L*), *MMP2* and *MMP3*^4–6,15^.

The Wnt gene family consists of 19 members in humans, all of which are highly conserved genes that regulate gene expression, cell behavior, cell adhesion and cell polarity^7^. WNT proteins, following their binding to the FZD and/or LRP5/6 receptors, activate the canonical WNT/β-catenin signaling pathway which in turn initiates a signaling transduction to several intracellular proteins to activate transcription of downstream genes^7^. Wnt/β-catenin signaling proteins participate in multiple developmental events during embryogenesis and adult tissue homeostasis. In bone, active Wnt/β-catenin signaling is critical, particularly for the differentiation of osteoblasts^16^. The activation of the canonical Wnt pathway in osteoblasts has been shown to upregulate OPG expression while downregulating RANKL expression, thereby suppressing bone resorption^17^. Moreover, defects or dysregulation of Wnt signaling are known to lead to various human skeletal diseases, such as osteogenesis imperfecta, osteoporosis, tetra-amelia (complete absence of all four limbs and other anomalies) and Robinow syndrome (short-limbed dwarfism and abnormalities in the head/face and external genitalia)^16^. However, the role of WNT genes/proteins in normal periapical tissues and in AP is not completely elucidated.

Our association findings point towards a likely role for *WNT3* and *WNT3A* towards increased AP risk and warrant additional studies using additional markers and other larger populations. The associated marker in *WNT3* (rs9890413), albeit located upstream of the gene, was not predicted to harbor any functional effects on gene/protein function however it may be in linkage disequilibrium with a true causal variant^18^. Fine-mapping genotyping efforts around the *WNT3* gene region may uncover the true causal variant.

Variations in *WNT3* have also been associated with alterations in bone mineral density and implicated in osteoporosis^19^, and with nonsyndromic cleft lip/palate, a common craniofacial anomaly and a defect of bone development^20^. Loss-of-function mutations in *WNT3* are also known to cause tetra-amelia, an extremely rare autosomal recessive congenital disorder characterized by the absence of all four limbs, and multiple malformations in the body including the face, skull and reproductive organs^21^. *WNT3A* is also known to be involved in craniofacial development^16^. Polymorphisms in *WNT3A* were associated with oral squamous cell carcinoma; *WNT3A* mRNA expression levels were also markedly higher in oral cancer tissues in comparison to normal mucosa^22^. Similar to *WNT3*, *WNT3A* rs1745420 has also been associated with non-syndromic cleft lip/palate^23^. These findings strengthen the role of these genes in bone-related conditions and disorders, including AP. Further, *WNT3A* rs1745420 may have a regulatory role on gene expression/function, due to our observations of an increased transcriptional activity (1.4-fold) in the presence of the alternate allele G when compared to the ancestral allele C. These findings also suggest that the ancestral allele C may play a role in reducing translation and/or stability of the mRNA^24^. rs1745420 is located next to several miRNA binding sites in the *WNT3A* gene (Supplementary Figs. S1 and S2) and may contribute to the modulation of gene expression post-transcriptionally^25^.

Our findings of differential expression of WNT proteins in AP tissues further supports a role for these molecules in AP development. In the present study, *WNT3*, *WNT3A* and *WNT5A* expression was significantly higher in AP lesions when compared to control tissues. Interestingly, *WNT3A* and *WNT5A* expression was also upregulated in chronic periodontal disease tissues in comparison to healthy tissues^26,27^. Expression of *WNT5A* was upregulated in inflammatory systemic diseases, such as rheumatoid arthritis^28^, and also in AP tissues, in which the levels of expression of WNT5A increased with the severity of inflammation^9^. Further, *WNT5A* mRNA levels varied in a dose-depend manner when osteoblasts were exposed to *Porphyromonas endodontalis* lipopolysaccharide (LPS)^29^. These observations of increased *WNT3A* and *WNT5A* expression in active lesions supports their involvement in AP lesion maintenance and/or progression.

We also assessed for potential correlations between the expression of the studied WNT genes with *SERPINB1*, *TIMP1*, and *COL1A1*, known markers for wound healing and bone repair^30^. Significant inverse correlations were observed between the expression of *WNT3A* and *WNT5A* with *SERPINB1*, a potent inhibitor of neutrophil elastase secreted by neutrophils and involved in tissue destruction^31^. This suggests that increased expression of *WNT3A* and *WNT5A* may impact inhibition of neutrophil elastase thereby contributing to tissue destruction. Similarly, inverse correlations were found between the expression of *WNT3A* and *WNT5A* with *COL1A1*, which is an abundant and key molecule of the bone matrix^32^. *WNT3* was also inversely correlated with *TIMP1* expression. *TIMP1* is a matrix metalloproteinase inhibitor with a critical role in bone remodeling and homeostasis; imbalances in TIMP gene expression may be related to increased or decreased bone resorption^33^. Interestingly, previous studies showed that *SERPINB1, TIMP1* and *COL1A1* were upregulated in AP lesions^30,34^. The expression of *TIMP1* and *COL1A1* was significantly higher in inactive AP lesions when compared to active lesions^30^. Similarly, *TIMP1* was highly expressed in asymptomatic AP lesions, corroborating our findings^35^.

The identification of key molecules for AP has the potential to improve currently available therapies. Recently, *in vitro* studies have shown the potential therapeutic use of WNT/β-catenin pathway molecules in AP treatment^8,11^. The expression of Dickkopf-1 (Dkk1), an antagonist of the WNT signaling pathway, was detected in induced rat periapical lesions and implicated in the inflammatory response and associated bone resorption^10^. Dkk1 facilitates osteoclastogenesis by increasing expression of Rankl and decreasing the expression of Opg^10^. Further, the use of a Dkk1 inhibitor in a rat AP model restored bone loss, indicating that this might be a potential therapeutic target for AP^11^. Transient treatment with lithium chloride (LiCl), an inhibitor of GSK3β, also resulted in bone formation and repair of induced AP lesions in rats, albeit continuous administration of the drug resulted in delayed bone repair^8^.

Overall the findings of the present study provide additional insights into the modulatory role of Wnt genes during AP. Among the limitations of this study are the limited sample size and the focused approach on a given gene family. In contrast, a strength of the present study is the thorough phenotypic characterization of the study populations, which in turn increase confidence in the results obtained. One can argue that a hypothesis-free approach, like genome-wide association study or GWAS, would be a better approach for an association study, but so far, there is no proof that this approach might overcome issues of all genetic studies, such as epigenetic, cytogenetic and environmental causes^36^. Furthermore, it has been shown that expected associations might be neglected if a strict correction for multiple testing is applied when testing for several variants simultaneously^37^. Additional studies with different populations need to be done to confirm the association between WNT genes and AP development. Considering that AP is a multifactorial disease, involving endodontic microbiota and host response^1^, gene polymorphisms may be among the players influencing AP development.

In summary, AP derives jointly from host and microbial factors. Our study shows that WNT genes may have a modulatory role in AP development and suggest *WNT3* and *WNT3A* as plausible contributor genes for AP. Dysregulation of contributor genes, as a result of genetic polymorphisms of the host, may influence destructive and/or remodeling events within the bone tissue. Identification of novel AP genes will elucidate the role of the host response in disease predisposition and pathogenesis and contribute towards improved treatment modalities.

## Methods

### Study populations and samples

This study followed the STROBE guidelines for human clinical studies. The Institutional Review Boards at the University of Pittsburgh (Project 0606091), University of São Paulo (Project 115/2009), Brazil, and the University of Texas Health Science Center at Houston (Project HSC-DB-12-0280) approved this study. The methods were carried out in accordance with the guidelines and regulations. Data extracted from electronic patient records and linked to DNA samples were provided in de-identified format. All individuals provided written informed consent agreeing to their participation in genetic studies. Three distinct datasets obtained at these institutions were used, as follows:

- Genetic association analysis: clinical/demographic history and saliva samples of individuals with and without AP were obtained at the University of Texas Health Science Center at Houston Graduate Endodontic Clinic and from the University of Pittsburgh Dental Registry and DNA Repository (DRDR)^38^. In brief, all individuals had to present one tooth showing a deep caries lesion (involving at least 2/3 of the dentin depth). Individuals received standard-of-care clinical endodontic diagnostic testing (thermal and electric pulp vitality tests, palpation and percussion tests) for evaluation of pulpal and periapical status^39^. The presence of a periapical lesion was determined by evaluation of individual periapical radiographs, as a periapical rarefaction characterized radiographically by the disappearance of the periodontal ligament space and discontinuity of the lamina dura^40^. Only AP lesions >3 mm were considered for this study. Based on clinical and radiographic findings, individuals were categorized as AP cases if in addition to deep caries, a pulpal diagnosis of asymptomatic irreversible pulpitis or pulp necrosis, and a periapical diagnosis of asymptomatic/ symptomatic AP or chronic apical abscess, and a periapical lesion ≥3 mm in diameter was found. Controls were individuals with deep caries, for which diagnostic testing confirmed a pulpal diagnosis of vital pulp tissues and/or irreversible pulpitis, and a periapical diagnosis of normal apical tissues (no AP lesion) or obvious widening of the periodontal ligament (PDL). The Pittsburgh dataset consisted of 109 AP cases and 155 controls (aged 20–60 years), whereas the Houston dataset had 79 cases and 75 controls (aged 18–65 years). Case and control groups were matched by age, sex, tobacco or alcohol usage.
- Gene expression analysis: discarded AP tissue samples were collected from individuals (n = 110, aged 19–61 years) referred to apical surgery or extraction after conventional root canal treatment had failed, at the University of Texas Health Science Center at Houston Dental Clinics and at the University of São Paulo (from 2009 to 2018). Upon collection, each sample was divided into 2 fragments of similar size and stored in 10% formalin or rinsed in PBS and immediately frozen. Samples stored in 10% formalin were submitted to histopathological analysis; frozen samples were used for gene expression analysis. All AP samples were histologically diagnosed as apical granulomas with or without epithelium (72%) and radicular cysts (28%). The clinical diagnosis included previously endodontically treated teeth (100%) with asymptomatic apical periodontitis (43%), symptomatic apical periodontitis (19%), and chronic apical abscess (38%). Healthy periodontal ligament tissues were collected from individuals referred extraction of premolars for orthodontic reasons (n = 26, aged 19–24 years) and used as control tissues.

Exclusion criteria for both analysis in this study included history of systemic conditions such as diabetes or other hormonal alterations related to exacerbated or uncontrolled inflammatory responses, and medical conditions requiring the use of systemic modifiers of bone metabolism or other assisted drug therapy (i.e., systemic antibiotics, anti-inflammatory, hormonal therapy) during the last 6 months before the study, preexisting conditions such as periodontal disease, and pregnant/ lactating women.

### Selection of contributor genes

We selected eleven SNPs in/nearby *WNT3*, *WNT3A*, *WNT5A*, *WNT8A*, *WNT9B* and *WNT11* genes (Table 1). We prioritize the SNPs to be investigated in our study based on: (1) previous published reports, (2) their locations within their respective genes, (3) their potential functional consequences (i.e., located in regulatory regions such as promoter, 3′ UTR, exon, or exon/intron boundaries), and/or (4) considered tag-SNPs as surrogates for the linkage disequilibrium blocks surrounding the contributor gene^12^. We used information available at the NCBI dbSNP (http://www.ncbi.nlm.gov/SNP/) and 1000 Genomes (http://www.1000genomes.org) databases to select polymorphisms.

### Genotyping

Genomic DNA was extracted from saliva using established protocols. Genotypes were generated using Taqman chemistry^41^. Reactions were carried out in 5-μL volumes in a ViiA7 Sequence Detection System (Applied Biosystems, Foster City, CA). In order to ensure quality control of genotyping reactions, a non-template control (water instead of DNA) was used as negative control and a DNA sample of known genotype as positive control. Genotype calls were automated using the TaqMan Genotyper Software (Applied Biosystems), and only automatic calls with a > 95% call rate were considered.

### *In Silico* bioinformatic analysis of SNV function

We used the FuncPred tool on the SNPinfo web server (https://snpinfo.niehs.nih.gov/snpinfo/snpfunc.html) and TargetScan (http://www.targetscan.org) for *in silico* prediction of SNV function for the associated *WNT3* rs9890413 and *WNT3A* rs1745420.

### Luciferase reporter assay

A reporter construct with 51 base pairs containing the *WNT3A* rs174520C/G variant was cloned downstream of the luciferase reporter gene of a 3′ UTR Reporter Vector (Active Motif, Carlsbad, CA), which contained RenSP, an optimized *Renilla* luminescent reporter gene. Cloning and sequence validation were obtained using Switchgear genomics custom cloning service (Active Motif). Amplicons sequences were: CACACCTGGCCTGCAAGGGGACCTT**C**GGCTCTCCACCCAGATGGCCCCCTG (allele C), and CACACCTGGCCTGCAAGGGGACCTT**G**GGCTCTCCACCCAGATGGCCCCCTG (allele G). Empty 3′ UTR vector (S890005, Active Motif) was used as positive control for transfection.

HEK293T cells (ATCC CRL-11268, ATCC, Manassas, VA) were cultured in Dulbecco’s Modified Eagle’s Medium supplemented with 10% fetal bovine serum and 1% penicillin/streptomycin at 37°C in 5% CO_2_ atmosphere. Cells were seeded at a density of 15,000 cells/well in 96-well plates (Corning, Kennebunk, ME), cultured to 80% confluence, transfected with 50 ng of each construct using FuGENE HD (Promega, Madison, WI) in OptiMEM medium (Life Technologies, Grand Island, NY) and cultured for 48 hours. Cells were lysed at −80°C overnight, thawed and incubated with 100 uL/well of LightSwitch reagent (Active Motif) for 30 minutes at room temperature. Luciferase activity was measured in a Tecan Infinite 200 Pro reader (Tecan, San Jose, CA) and normalized to *Renilla* luciferase activity levels. Three independent experiments were conducted in sextuplicates for each construct. Luciferase activity was recorded as arbitrary units of fluoresce (AU). Data is presented as mean ± standard deviation of three independent experiments.

### Gene expression assays

Total RNA was extracted using RNAeasy kit (Qiagen, Valencia, CA). RNA quality was analyzed using 2100 Bioanalyzer (Agilent Technologies, Santa Clara, CA), and complementary DNA was synthesized using 3 μg RNA/sample as a template in a reverse-transcription reaction (QuantiTectRT Kit, Qiagen). AP tissues were first categorized into active/progressive (n = 40; RANKL > OPG) or inactive/stable (n = 70; RANKL ≤ OPG) based on RANKL/OPG expression ratios as previously described^13^. Next, we evaluated the mRNA levels of *WNT3*, *WNT3A*, *WNT5A*, *WNT9B*, *SERPINB1*, *TIMP1* and *COL1A1* in the active and inactive AP lesions using TaqMan gene expression assays (Invitrogen, Carlsbad, CA) in a ViiA7 Real Time PCR instrument (Life Technologies, Carlsbad, CA). Experiments were run in triplicates and repeated twice.

### Statistical analysis

Power calculations were performed using GAS Power Calculator (http://csg.sph.umich.edu/abecasis/cats/gas_power_calculator/index.html) and indicate that the proposed sample size will provide ~97% statistical power to detect an association with an alpha of 0.05, under a multiplicative disease model, prevalence and disease allele frequency of 25% (Supplementary Fig. S2).

We tested for deviation from Hardy-Weinberg equilibrium (HWE) using PLINK v.1.05 software^42^. Allele and genotype frequencies for each SNP in cases and controls were compared using Fisher Exact tests as implemented in PLINK. Haplotype association analyses were performed for SNPs in chromosome 1 (*WNT3A*) and 17 (*WNT3* and *WNT9B*) using ‘haplotype-based case-control test’ function. Bonferroni correction was used to adjust for multiple testing (0.05/11 SNPs) and α ≤ 0.005 indicated statistically significant differences between groups.

For the luciferase assay, differences between groups were tested using Kruskal-Wallis and post hoc Dunn’s test, as implemented in GraphPad Prism 7 (GraphPad, La Jolla, CA).

Expression levels of target genes relative to the expression of housekeeping genes (*GAPDH* and β-actin) in each sample were analyzed using the 2−^ΔΔ^Ct method^43^. Statistical differences between groups were assessed using Mann-Whitney test and linear regression. Analyses were performed in GraphPad Prism 7. P ≤ 0.05 indicated statistically significant differences between groups.

## Supplementary information

Supplementary Info

## Data Availability

Individual level data for any of the experiments described in the study may be available upon request.
