# Supplementary material for: WNT gene polymorphisms and predisposition to apical periodontitis
Source: Sci Rep. 2019 Dec 12;9:18980. doi: 10.1038/s41598-019-55293-6 (PMC6908593; doi:10.1038/s41598-019-55293-6)
Supplement: Supplementary file 1 — Supplementary Info [file 41598_2019_55293_MOESM1_ESM.docx]

**WNT gene polymorphisms and predisposition to apical periodontitis**

Letícia Chaves de Souza, Franco Cavalla, Lorena Maili, Gustavo Pompermaier Garlet, Alexandre R. Vieira, Renato Menezes Silva & Ariadne Letra*

**Supplemental files**

Supplementary Table S1. Allelic and genotypic association results for the Houston population

| **Gene** | **SNP Id** | **MAF CEU^a,b^** | **MAF (cases)** | **MAF (control)** | **Test** | **Alleles** | **Frequency (cases)** | **Frequency (controls)** | **P value^c^** |
| --- | --- | --- | --- | --- | --- | --- | --- | --- | --- |
| *WNT3A* | rs708111 |  |  |  | Genotypic | GG/GA/AA | 15/42/22 | 14/37/24 | 0.85 |
|  |  | 0.45(G) | 0.46(G) | 0.43(G) | Allelic | G/A | 72/86 | 65/85 | 0.69 |
|  |  |  |  |  | Dominant | GG+GA/AA | 57/22 | 51/24 | 0.57 |
|  |  |  |  |  | Recessive | GG/GA+AA | 15/64 | 14/61 | 0.96 |
|  | rs3094912 |  |  |  | Genotypic | AA/AT/TT | 15/42/22 | 14/36/24 | 0.81 |
|  |  | 0.45(T) | 0.46(A) | 0.43(A) | Allelic | A/T | 72/86 | 64/84 | 0.68 |
|  |  |  |  |  | Dominant | AA+AT/TT | 57/22 | 50/24 | 0.54 |
|  |  |  |  |  | Recessive | AA/AT+TT | 15/64 | 14/60 | 0.99 |
|  | rs752107 |  |  |  | Genotypic | TT/TC/CC | 8/34/37 | 2/36/37 | NA |
|  |  | 0.29(T) | 0.32(T) | 0.27(T) | Allelic | T/C | 50/108 | 40/110 | 0.34 |
|  |  |  |  |  | Dominant | TT+TC/CC | 42/37 | 38/37 | NA |
|  |  |  |  |  | Recessive | TT/TC+CC | 8/71 | 2/73 | NA |
|  | rs1745420 |  |  |  | Genotypic | CC/CG/GG | 10/26/42 | 16/33/25 | 0.04 |
|  |  | 0.22(C) | 0.29(C) | 0.44(C) | Allelic | C/G | 46/110 | 65/83 | 0.009 |
|  |  |  |  |  | Dominant | CC+CG/GG | 36/42 | 49/25 | 0.01 |
|  |  |  |  |  | Recessive | CC/CG+GG | 10/68 | 16/58 | 0.15 |
| *WNT8A* | rs2040862 |  |  |  | Genotypic | TT/TC/CC | 0/18/61 | 1/11/62 | NA |
|  |  | 0.15(T) | 0.11(T) | 0.09(T) | Allelic | T/C | 18/140 | 13/135 | 0.45 |
|  |  |  |  |  | Dominant | TT+TC/CC | 18/61 | 12/62 | NA |
|  |  |  |  |  | Recessive | TT/TC+CC | 0/79 | 1/73 | NA |
| *WNT11* | rs1533767 |  |  |  | Genotypic | AA/AG/GG | 4/27/46 | 3/31/40 | NA |
|  |  | 0.22(A) | 0.23(A) | 0.25(A) | Allelic | A/G | 35/119 | 37/111 | 0.64 |
|  |  |  |  |  | Dominant | AA+AG/GG | 31/46 | 34/40 | NA |
|  |  |  |  |  | Recessive | AA/AG+GG | 4/73 | 3/71 | NA |
| *WNT3* | rs199498 |  |  |  | Genotypic | CC/CT/TT | 10/26/43 | 5/26/43 | 0.47 |
|  |  | 0.20(C) | 0.29(C) | 0.24(C) | Allelic | C/T | 46/112 | 36/112 | 0.34 |
|  |  |  |  |  | Dominant | CC+CT/TT | 36/43 | 31/43 | 0.65 |
|  |  |  |  |  | Recessive | CC/CT+TT | 10/69 | 5/69 | 0.22 |
|  | rs111769 |  |  |  | Genotypic | TT/TC/CC | 5/35/39 | 6/27/42 | 0.57 |
|  |  | 0.41(T) | 0.28(T) | 0.26(T) | Allelic | T/C | 45/113 | 39/111 | 0.62 |
|  |  |  |  |  | Dominant | TT+TC/CC | 40/39 | 33/42 | 0.41 |
|  |  |  |  |  | Recessive | TT/TC+CC | 5/74 | 6/69 | 0.69 |
|  | rs9890413 |  |  |  | Genotypic | GG/GA/AA | 8/36/35 | 5/29/40 | 0.44 |
|  |  | 0.36(G) | 0.33(G) | 0.26(G) | Allelic | G/A | 52/106 | 39/109 | 0.21 |
|  |  |  |  |  | Dominant | GG+GA/AA | 44/35 | 34/40 | 0.23 |
|  |  |  |  |  | Recessive | GG/GA+AA | 8/71 | 5/69 | 0.45 |
| *WNT9B* | rs2165846 |  |  |  | Genotypic | AA/AG/GG | 10/33/36 | 18/33/22 | 0.07 |
|  |  | 0.40(G) | 0.33(A) | 0.47(A) | Allelic | A/G | 53/105 | 69/77 | 0.01 |
|  |  |  |  |  | Dominant | AA+AG/GG | 43/36 | 51/22 | 0.05 |
|  |  |  |  |  | Recessive | AA/AG+GG | 10/69 | 18/55 | 0.06 |

^a^According to Ensembl GRCh38.p12 assembly (June 21, 2019). ^b^Minor allele frequency (MAF) in CEU population. ^c^Fisher exact test, Bonferroni correction, significant if α≤0.005. Italic font means 0.006≤P≤0.009. SNP rs566926 was out of Hardy-Weinberg equilibrium (HWE), therefore, was excluded from the analysis.

Supplementary Table S2. Allelic and genotypic association results for the Pittsburgh population

| **Gene** | **SNP Id** | **MAF CEU^a,b^** | **MAF (cases)** | **MAF (control)** | **Test** | **Alleles** | **Frequency (cases)** | **Frequency (controls)** | **P value^c^** |
| --- | --- | --- | --- | --- | --- | --- | --- | --- | --- |
| *WNT3A* | rs708111 |  |  |  | Genotypic | AA/AG/GG | 25/51/31 | 44/65/44 | 0.59 |
|  |  | 0.45(G) | 0.47(A) | 0.5(A) | Allelic | A/G | 101/113 | 153/153 | 0.53 |
|  |  |  |  |  | Dominant | AA+AG/GG | 76/31 | 109/44 | 0.97 |
|  |  |  |  |  | Recessive | AA/AG+GG | 25/82 | 44/109 | 0.33 |
|  | rs3094912 |  |  |  | Genotypic | TT/TA/AT | 27/51/29 | 44/64/45 | 0.64 |
|  |  | 0.45(T) | 0.49(T) | 0.50(T) | Allelic | T/A | 105/109 | 152/154 | 0.89 |
|  |  |  |  |  | Dominant | TT+TA/AA | 78/29 | 108/45 | 0.68 |
|  |  |  |  |  | Recessive | AA/AT+TT | 27/80 | 44/109 | 0.53 |
|  | rs752107 |  |  |  | Genotypic | TT/TC/CC | 6/48/54 | 17/57/76 | 0.22 |
|  |  | 0.29(T) | 0.28(T) | 0.30(T) | Allelic | T/C | 60/156 | 91/209 | 0.53 |
|  |  |  |  |  | Dominant | TT+TC/CC | 54/54 | 74/76 | 0.92 |
|  |  |  |  |  | Recessive | TT/TC+CC | 6/102 | 17/133 | 0.11 |
|  | rs1745420 |  |  |  | Genotypic | CC/CG/GG | 6/27/72 | 5/33/112 | 0.47 |
|  |  | 0.22(C) | 0.19(C) | 0.14(C) | Allelic | C/G | 39/171 | 43/257 | 0.20 |
|  |  |  |  |  | Dominant | CC+CG/GG | 33/72 | 38/112 | 0.29 |
|  |  |  |  |  | Recessive | CC/CG+GG | 6/99 | 5/145 | 0.36 |
| *WNT11* | rs1533767 |  |  |  | Genotypic | AA/AG/GG | 2/37/67 | 9/54/86 | NA |
|  |  | 0.22(A) | 0.19(A) | 0.24(A) | Allelic | A/G | 41/171 | 72/226 | 0.20 |
|  |  |  |  |  | Dominant | AA+AG/GG | 39/67 | 63/86 | NA |
|  |  |  |  |  | Recessive | AA/AG+GG | 2/104 | 9/140 | NA |
| *WNT3* | rs199498 |  |  |  | Genotypic | CC/CT/TT | 8/37/59 | 7/45/87 | 0.55 |
|  |  | 0.20(C) | 0.25(C) | 0.21(C) | Allelic | C/T | 53/155 | 59/219 | 0.27 |
|  |  |  |  |  | Dominant | CC+CT/TT | 45/59 | 52/87 | 0.36 |
|  |  |  |  |  | Recessive | CC/CT+TT | 8/96 | 7/132 | 0.39 |
|  | rs111769 |  |  |  | Genotypic | TT/TC/CC | 9/45/47 | 21/68/51 | 0.18 |
|  |  | 0.41(T) | 0.31(T) | 0.39(T) | Allelic | T/C | 63/139 | 110/170 | 0.07 |
|  |  |  |  |  | Dominant | TT+TC/CC | 54/47 | 89/51 | 0.11 |
|  |  |  |  |  | Recessive | TT/TC+CC | 9/92 | 21/119 | 0.16 |
|  | rs9890413 |  |  |  | Genotypic | GG/GA/AA | 14/49/35 | 12/51/71 | 0.03 |
|  |  | 0.36(G) | 0.39(G) | 0.28(G) | Allelic | G/A | 77/119 | 75/193 | 0.01 |
|  |  |  |  |  | Dominant | GG+GA/AA | 63/35 | 63/71 | *0.009* |
|  |  |  |  |  | Recessive | GG/GA+AA | 14/84 | 12/122 | 0.20 |
| *WNT9B* | rs2165846 |  |  |  | Genotypic | GG/GA/AA | 17/40/24 | 26/50/35 | 0.83 |
|  |  | 0.40(G) | 0.46(G) | 0.46(G) | Allelic | G/A | 74/88 | 102/120 | 0.96 |
|  |  |  |  |  | Dominant | GG+GA/AA | 57/24 | 76/35 | 0.78 |
|  |  |  |  |  | Recessive | GG/GA+AA | 17/64 | 26/85 | 0.69 |

^a^According to Ensembl GRCh38.p12 assembly (June 21, 2019). ^b^Minor allele frequency (MAF) in CEU population. ^c^Fisher exact test, Bonferroni correction, significant if α≤0.005. Italic font means 0.006≤P≤0.009. SNPs rs566926 and rs2040862 were out of HWE and excluded from analysis.

Supplementary Table S3. Allelic and genotypic association results for the combined populations

| **Gene** | **SNP Id** | **MAF CEU^a.b^** | **MAF (cases)** | **MAF (control)** | **Test** | **Alleles** | **Frequency (cases)** | **Frequency (controls)** | **P value^c^** |
| --- | --- | --- | --- | --- | --- | --- | --- | --- | --- |
| *WNT3A* | rs708111 |  |  |  | Genotypic | GG/GA/AA | 46/93/47 | 58/102/68 | 0.50 |
|  |  | 0.45(G) | 0.50(G) | 0.48(G) | Allelic | G/A | 185/187 | 218/238 | 0.58 |
|  |  |  |  |  | Dominant | GG+GA/AA | 139/47 | 160/68 | 0.30 |
|  |  |  |  |  | Recessive | GG/GA+AA | 46/140 | 58/170 | 0.87 |
|  | rs3094912 |  |  |  | Genotypic | AA/AT/TT | 44/93/49 | 59/100/68 | 0.48 |
|  |  | 0.45(T) | 0.49(A) | 0.48(A) | Allelic | A/T | 181/191 | 218/236 | 0.85 |
|  |  |  |  |  | Dominant | AA+AT/TT | 137/49 | 159/68 | 0.42 |
|  |  |  |  |  | Recessive | AA/AT+TT | 44/142 | 59/168 | 0.59 |
|  | rs752107 |  |  |  | Genotypic | TT/TC/CC | 14/82/91 | 19/93/113 | 0.85 |
|  |  | 0.29(T) | 0.29(T) | 0.29(T) | Allelic | T/C | 110/264 | 131/319 | 0.92 |
|  |  |  |  |  | Dominant | TT+TC/CC | 96/91 | 112/113 | 0.75 |
|  |  |  |  |  | Recessive | TT/TC+CC | 14/173 | 19/206 | 0.72 |
|  | rs1745420 |  |  |  | Genotypic | CC/CG/GG | 16/53/114 | 21/66/137 | 0.96 |
|  |  | 0.22(C) | 0.23(C) | 0.24(C) | Allelic | C/G | 85/281 | 108/340 | 0.77 |
|  |  |  |  |  | Dominant | CC+CG/GG | 69/114 | 87/137 | 0.81 |
|  |  |  |  |  | Recessive | CC/CG+GG | 16/167 | 21/203 | 0.82 |
| *WNT8A* | rs2040862 |  |  |  | Genotypic | TT/TC/CC | 1/42/91 | 1/45/113 | NA |
|  |  | 0.15(T) | 0.16(T) | 0.15(T) | Allelic | T/C | 44/224 | 47/271 | 0.59 |
|  |  |  |  |  | Dominant | TT+TC/CC | 43/91 | 46/113 | NA |
|  |  |  |  |  | Recessive | TT/TC+CC | 1/133 | 1/158 | NA |
| *WNT11* | rs1533767 |  |  |  | Genotypic | AA/AG/GG | 6/64/113 | 12/85/126 | 0.42 |
|  |  | 0.22(A) | 0.21(A) | 0.24(A) | Allelic | A/G | 76/290 | 109/337 | 0.21 |
|  |  |  |  |  | Dominant | AA+AG/GG | 70/113 | 97/126 | 0.29 |
|  |  |  |  |  | Recessive | AA/AG+GG | 6/177 | 12/211 | 0.31 |
| *WNT3* | rs199498 |  |  |  | Genotypic | CC/CT/TT | 18/63/102 | 12/71/130 | 0.25 |
|  |  | 0.20(C) | 0.27(C) | 0.22(C) | Allelic | C/T | 99/267 | 95/331 | 0.12 |
|  |  |  |  |  | Dominant | CC+CT/TT | 81/102 | 83/130 | 0.29 |
|  |  |  |  |  | Recessive | CC/CT+TT | 18/165 | 12/201 | 0.11 |
|  | rs111769 |  |  |  | Genotypic | TT/TC/CC | 14/80/86 | 27/95/93 | 0.27 |
|  |  | 0.41(T) | 0.3(T) | 0.35(T) | Allelic | T/C | 108/252 | 149/281 | 0.16 |
|  |  |  |  |  | Dominant | TT+TC/CC | 94/86 | 122/93 | 0.37 |
|  |  |  |  |  | Recessive | TT/TC+CC | 14/166 | 27/188 | 0.12 |
|  | rs9890413 |  |  |  | Genotypic | GG/GA/AA | 22/85/70 | 17/80/111 | 0.02 |
|  |  | 0.36(G) | 0.36(G) | 0.27(G) | Allelic | G/A | 129/225 | 114/302 | 0.007 |
|  |  |  |  |  | Dominant | GG+GA/AA | 107/70 | 97/111 | 0.007 |
|  |  |  |  |  | Recessive | GG/GA+AA | 22/155 | 17/191 | 0.17 |
| *WNT9B* | rs2165846 |  |  |  | Genotypic | AA/AG/GG | 34/73/53 | 53/83/48 | 0.18 |
|  |  | 0.40(G) | 0.44(A) | 0.51(A) | Allelic | A/G | 141/179 | 189/179 | 0.06 |
|  |  |  |  |  | Dominant | AA+AG/GG | 107/53 | 136/48 | 0.15 |
|  |  |  |  |  | Recessive | AA/AG+GG | 34/126 | 53/131 | 0.11 |

^a^According to Ensembl GRCh38.p12 assembly (June 21, 2019). ^b^Minor allele frequency (MAF) in CEU population. ^c^Fisher exact test, Bonferroni correction, significant if α≤0.005. Italic font means 0.006≤P≤0.009. SNP rs566926 was out of HWE and excluded from analysis.

Supplementary Table S4. Haplotype analysis results

| **Dataset** | **Gene** | **SNPs** | **Haplotype** | **Frequency of cases** | **Frequency of controls** | **P value^a^** |
| --- | --- | --- | --- | --- | --- | --- |
| Houston | *WNT3A* | rs708111 / rs3094912 | GA | 0.46 | 0.43 | 0.67 |
|  |  |  | AT | 0.54 | 0.57 | 0.67 |
|  |  | rs3094912 / rs752107 | TT | 0.32 | 0.26 | 0.26 |
|  |  |  | AC | 0.46 | 0.43 | 0.63 |
|  |  |  | TC | 0.23 | 0.31 | 0.09 |
|  |  | rs752107 / rs1745420 | TC | 0.06 | 0.05 | 0.66 |
|  |  |  | CC | 0.23 | 0.39 | **0.003** |
|  |  |  | TG | 0.25 | 0.21 | 0.42 |
|  |  |  | CG | 0.46 | 0.35 | 0.06 |
|  |  | rs708111/ rs3094912 / rs752107 | ATT | 0.32 | 0.27 | 0.33 |
|  |  |  | GAC | 0.46 | 0.43 | 0.63 |
|  |  |  | ATC | 0.23 | 0.31 | 0.12 |
|  |  | rs3094912 / rs752107 / rs1745420 | TTC | 0.06 | 0.04 | 0.47 |
|  |  |  | ACC | 0.06 | 0.12 | 0.05 |
|  |  |  | TCC | 0.17 | 0.28 | 0.03 |
|  |  |  | TTG | 0.25 | 0.22 | 0.45 |
|  |  |  | ACG | 0.40 | 0.30 | 0.09 |
|  |  |  | TCG | 0.06 | 0.03 | 0.38 |
|  |  | rs708111 / rs3094912 / rs752107 / rs1745420 | ATTC | 0.06 | 0.04 | 0.48 |
|  |  |  | GACC | 0.06 | 0.13 | 0.04 |
|  |  |  | ATCC | 0.17 | 0.27 | 0.04 |
|  |  |  | ATTG | 0.25 | 0.22 | 0.50 |
|  |  |  | GACG | 0.40 | 0.30 | 0.09 |
|  |  |  | ATCG | 0.06 | 0.03 | 0.36 |
|  | *WNT3* | rs199498 / rs111769 | CT | 0.11 | 0.07 | 0.25 |
|  |  |  | TT | 0.18 | 0.19 | 0.84 |
|  |  |  | CC | 0.19 | 0.17 | 0.81 |
|  |  |  | TC | 0.53 | 0.57 | 0.50 |
|  |  | rs111769 / rs9890413 | TG | 0.15 | 0.13 | 0.60 |
|  |  |  | CG | 0.18 | 0.14 | 0.29 |
|  |  |  | TA | 0.14 | 0.14 | 0.99 |
|  |  |  | CA | 0.53 | 0.60 | 0.24 |
|  |  | rs199498 / rs111769 / rs9890413 | CTG | 0.05 | 0.05 | 0.82 |
|  |  |  | TTG | 0.09 | 0.08 | 0.56 |
|  |  |  | CCG | 0.04 | 0.02 | 0.27 |
|  |  |  | TCG | 0.14 | 0.12 | 0.59 |
|  |  |  | CTA | 0.05 | 0.02 | 0.19 |
|  |  |  | TTA | 0.09 | 0.11 | 0.42 |
|  |  |  | CCA | 0.15 | 0.16 | 0.88 |
|  |  |  | TCA | 0.39 | 0.45 | 0.29 |
|  | *WNT3 / WNT9B* | rs9890413 / rs2165846 | GA | 0.12 | 0.12 | 0.95 |
|  |  |  | AA | 0.21 | 0.35 | *0.007* |
|  |  |  | GG | 0.21 | 0.15 | 0.15 |
|  |  |  | AG | 0.45 | 0.38 | 0.19 |
|  | *WNT3 / WNT9B* | rs111769 / rs9890413 / rs2165846 | TGA | 0.08 | 0.08 | 0.86 |
|  |  |  | CGA | 0.04 | 0.04 | 0.97 |
|  |  |  | TAA | 0.08 | 0.10 | 0.55 |
|  |  |  | CAA | 0.13 | 0.25 | *0.007* |
|  |  |  | TGG | 0.06 | 0.05 | 0.54 |
|  |  |  | CGG | 0.14 | 0.10 | 0.22 |
|  |  |  | TAG | 0.06 | 0.03 | 0.34 |
|  |  |  | CAG | 0.40 | 0.35 | 0.36 |
|  | *WNT3 / WNT9B* | rs199498 / rs111769 / rs9890413 / rs2165846 | CTGA | 0.05 | 0.04 | 0.71 |
|  |  |  | TTGA | 0.04 | 0.04 | 0.93 |
|  |  |  | TCGA | 0.02 | 0.03 | 0.66 |
|  |  |  | CTAA | 0.01 | 0.01 | 0.94 |
|  |  |  | TTAA | 0.07 | 0.08 | 0.53 |
|  |  |  | CCAA | 0.007 | 0.02 | 0.41 |
|  |  |  | TCAA | 0.12 | 0.24 | *0.009* |
|  |  |  | CTGG | 0.01 | 0.01 | 0.84 |
|  |  |  | TTGG | 0.05 | 0.03 | 0.43 |
|  |  |  | CCGG | 0.03 | 0.01 | 0.39 |
|  |  |  | TCGG | 0.12 | 0.09 | 0.43 |
|  |  |  | CTAG | 0.03 | 0.006 | 0.07 |
|  |  |  | TTAG | 0.02 | 0.03 | 0.70 |
|  |  |  | CCAG | 0.14 | 0.14 | 0.95 |
|  |  |  | TCAG | 0.27 | 0.21 | 0.22 |
| Pittsburgh | *WNT3A* | rs708111 / rs3094912 | AT | 0.45 | 0.47 | 0.64 |
|  |  |  | GT | 0.04 | 0.02 | 0.32 |
|  |  |  | AA | 0.02 | 0.03 | 0.59 |
|  |  |  | GA | 0.49 | 0.48 | 0.76 |
|  |  | rs3094912 / rs752107 | TT | 0.25 | 0.29 | 0.32 |
|  |  |  | AT | 0.02 | 0.01 | 0.25 |
|  |  |  | TC | 0.24 | 0.20 | 0.36 |
|  |  |  | AC | 0.49 | 0.49 | 0.89 |
|  |  | rs752107 / rs1745420 | CC | 0.18 | 0.14 | 0.25 |
|  |  |  | TG | 0.28 | 0.30 | 0.58 |
|  |  |  | CG | 0.54 | 0.55 | 0.74 |
|  |  | rs708111/ rs3094912 / rs752107 | ATT | 0.24 | 0.29 | 0.25 |
|  |  |  | GTT | 0.02 | 0.01 | 0.26 |
|  |  |  | ATC | 0.22 | 0.19 | 0.40 |
|  |  |  | GTC | 0.02 | 0.01 | 0.71 |
|  |  |  | AAC | 0.01 | 0.02 | 0.59 |
|  |  |  | GAC | 0.48 | 0.48 | 0.88 |
|  |  | rs3094912 / rs752107 / rs1745420 | TCC | 0.17 | 0.13 | 0.19 |
|  |  |  | ACC | 0.01 | 0.01 | 0.89 |
|  |  |  | TTG | 0.25 | 0.29 | 0.35 |
|  |  |  | ATG | 0.02 | 0.01 | 0.31 |
|  |  |  | TCG | 0.07 | 0.08 | 0.72 |
|  |  |  | ACG | 0.48 | 0.49 | 0.85 |
|  |  | rs708111 / rs3094912 / rs752107 / rs1745420 | ATCC | 0.16 | 0.12 | 0.21 |
|  |  |  | ATTG | 0.25 | 0.29 | 0.29 |
|  |  |  | ATCG | 0.06 | 0.07 | 0.78 |
|  |  |  | GTCG | 0.02 | 0.01 | 0.69 |
|  |  |  | AACG | 0.01 | 0.02 | 0.28 |
|  |  |  | GACG | 0.50 | 0.48 | 0.70 |
|  | *WNT3* | rs199498 / rs111769 | CT | 0.10 | 0.10 | 0.84 |
|  |  |  | TT | 0.21 | 0.30 | 0.04 |
|  |  |  | CC | 0.15 | 0.11 | 0.20 |
|  |  |  | TC | 0.54 | 0.50 | 0.41 |
|  |  | rs111769 / rs9890413 | TG | 0.15 | 0.18 | 0.43 |
|  |  |  | CG | 0.24 | 0.10 | **0.00008** |
|  |  |  | TA | 0.15 | 0.22 | 0.05 |
|  |  |  | CA | 0.46 | 0.50 | 0.44 |
|  |  | rs199498 / rs111769 / rs9890413 | CTG | 0.07 | 0.07 | 0.93 |
|  |  |  | TTG | 0.09 | 0.10 | 0.68 |
|  |  |  | CCG | 0.02 | 0.005 | 0.16 |
|  |  |  | TCG | 0.21 | 0.10 | **0.0005** |
|  |  |  | CTA | 0.04 | 0.03 | 0.88 |
|  |  |  | TTA | 0.11 | 0.19 | 0.03 |
|  |  |  | CCA | 0.12 | 0.10 | 0.43 |
|  |  |  | TCA | 0.33 | 0.40 | 0.13 |
|  | *WNT3 / WNT9B* | rs9890413 / rs2165846 | GG | 0.22 | 0.12 | 0.01 |
|  |  |  | AG | 0.23 | 0.33 | 0.03 |
|  |  |  | GA | 0.17 | 0.16 | 0.72 |
|  |  |  | AA | 0.37 | 0.38 | 0.84 |
|  | *WNT3 / WNT9B* | rs111769 / rs9890413 / rs2165846 | TGG | 0.02 | 0.03 | 0.47 |
|  |  |  | CGG | 0.20 | 0.09 | **0.0002** |
|  |  |  | TAG | 0.05 | 0.10 | 0.07 |
|  |  |  | CAG | 0.18 | 0.23 | 0.16 |
|  |  |  | TGA | 0.12 | 0.14 | 0.54 |
|  |  |  | CGA | 0.04 | 0.02 | 0.23 |
|  |  |  | TAA | 0.10 | 0.13 | 0.37 |
|  |  |  | CAA | 0.27 | 0.26 | 0.65 |
|  | *WNT3 / WNT9B* | rs199498 / rs111769 / rs9890413 / rs2165846 | TTGG | 0.02 | 0.03 | 0.55 |
|  |  |  | TCGG | 0.20 | 0.09 | **0.0009** |
|  |  |  | CTAG | 0.02 | 0.02 | 0.66 |
|  |  |  | TTAG | 0.04 | 0.08 | 0.08 |
|  |  |  | CCAG | 0.05 | 0.04 | 0.90 |
|  |  |  | TCAG | 0.14 | 0.19 | 0.15 |
|  |  |  | CTGA | 0.06 | 0.06 | 0.88 |
|  |  |  | TTGA | 0.06 | 0.07 | 0.69 |
|  |  |  | TCGA | 0.04 | 0.02 | 0.34 |
|  |  |  | CTAA | 0.02 | 0.01 | 0.69 |
|  |  |  | TTAA | 0.09 | 0.12 | 0.23 |
|  |  |  | CCAA | 0.08 | 0.04 | 0.08 |
|  |  |  | TCAA | 0.20 | 0.22 | 0.70 |
| Combined | *WNT3A* | rs708111 / rs3094912 | GA | 0.48 | 0.46 | 0.66 |
|  |  |  | AA | 0.01 | 0.02 | 0.30 |
|  |  |  | GT | 0.02 | 0.02 | 0.68 |
|  |  |  | AT | 0.49 | 0.50 | 0.77 |
|  |  | rs3094912 / rs752107 | AT | 0.01 | 0.01 | 0.55 |
|  |  |  | TT | 0.28 | 0.28 | 0.96 |
|  |  |  | AC | 0.47 | 0.47 | 0.95 |
|  |  |  | TC | 0.23 | 0.24 | 0.86 |
|  |  | rs752107 / rs1745420 | TC | 0.03 | 0.02 | 0.31 |
|  |  |  | CC | 0.20 | 0.22 | 0.46 |
|  |  |  | TG | 0.26 | 0.27 | 0.87 |
|  |  |  | CG | 0.50 | 0.49 | 0.67 |
|  |  | rs708111/ rs3094912 / rs752107 | ATT | 0.28 | 0.28 | 0.92 |
|  |  |  | GAC | 0.48 | 0.46 | 0.67 |
|  |  |  | AAC | 0.01 | 0.02 | 0.33 |
|  |  |  | GTC | 0.01 | 0.01 | 0.94 |
|  |  |  | ATC | 0.23 | 0.23 | 0.90 |
|  |  | rs3094912 / rs752107 / rs1745420 | TTC | 0.03 | 0.02 | 0.24 |
|  |  |  | ACC | 0.03 | 0.05 | 0.23 |
|  |  |  | TCC | 0.17 | 0.17 | 0.88 |
|  |  |  | ATG | 0.01 | 0.01 | 0.56 |
|  |  |  | TTG | 0.25 | 0.26 | 0.68 |
|  |  |  | ACG | 0.44 | 0.42 | 0.62 |
|  |  |  | TCG | 0.06 | 0.06 | 0.98 |
|  |  | rs708111 / rs3094912 / rs752107 / rs1745420 | ATTC | 0.03 | 0.02 | 0.28 |
|  |  |  | GACC | 0.03 | 0.05 | 0.16 |
|  |  |  | ATCC | 0.17 | 0.17 | 0.89 |
|  |  |  | ATTG | 0.25 | 0.27 | 0.65 |
|  |  |  | GACG | 0.45 | 0.42 | 0.34 |
|  |  |  | AACG | 0.01 | 0.02 | 0.25 |
|  |  |  | ATCG | 0.06 | 0.06 | 0.91 |
|  | *WNT3* | rs199498 / rs111769 | CT | 0.10 | 0.09 | 0.41 |
|  |  |  | TT | 0.20 | 0.26 | 0.05 |
|  |  |  | CC | 0.16 | 0.13 | 0.21 |
|  |  |  | TC | 0.53 | 0.52 | 0.76 |
|  |  | rs111769 / rs9890413 | TG | 0.15 | 0.16 | 0.66 |
|  |  |  | CG | 0.21 | 0.11 | **0.0002** |
|  |  |  | TA | 0.14 | 0.19 | 0.08 |
|  |  |  | CA | 0.49 | 0.53 | 0.26 |
|  |  | rs199498 / rs111769 / rs9890413 | CTG | 0.06 | 0.06 | 0.98 |
|  |  |  | TTG | 0.09 | 0.10 | 0.92 |
|  |  |  | CCG | 0.03 | 0.01 | 0.07 |
|  |  |  | TCG | 0.18 | 0.11 | **0.002** |
|  |  |  | CTA | 0.04 | 0.03 | 0.36 |
|  |  |  | TTA | 0.10 | 0.16 | 0.02 |
|  |  |  | CCA | 0.14 | 0.12 | 0.51 |
|  |  |  | TCA | 0.35 | 0.42 | 0.08 |
|  | *WNT3 / WNT9B* | rs9890413 / rs2165846 | GA | 0.15 | 0.14 | 0.89 |
|  |  |  | AA | 0.29 | 0.37 | 0.03 |
|  |  |  | GG | 0.21 | 0.13 | **0.004** |
|  |  |  | AG | 0.35 | 0.35 | 0.82 |
|  | *WNT3 / WNT9B* | rs111769 / rs9890413 / rs2165846 | TGA | 0.10 | 0.12 | 0.55 |
|  |  |  | CGA | 0.04 | 0.03 | 0.28 |
|  |  |  | TAA | 0.09 | 0.12 | 0.21 |
|  |  |  | CAA | 0.21 | 0.25 | 0.14 |
|  |  |  | TGG | 0.05 | 0.05 | 0.98 |
|  |  |  | CGG | 0.17 | 0.08 | **0.0003** |
|  |  |  | TAG | 0.05 | 0.07 | 0.35 |
|  |  |  | CAG | 0.29 | 0.28 | 0.96 |
|  | *WNT3 / WNT9B* | rs199498 / rs111769 / rs9890413 / rs2165846 | CTGA | 0.05 | 0.06 | 0.97 |
|  |  |  | TTGA | 0.05 | 0.06 | 0.68 |
|  |  |  | TCGA | 0.03 | 0.02 | 0.60 |
|  |  |  | CTAA | 0.01 | 0.01 | 0.83 |
|  |  |  | TTAA | 0.08 | 0.11 | 0.13 |
|  |  |  | CCAA | 0.04 | 0.03 | 0.34 |
|  |  |  | TCAA | 0.17 | 0.23 | 0.04 |
|  |  |  | TTGG | 0.04 | 0.04 | 0.89 |
|  |  |  | CCGG | 0.02 | 0.006 | 0.13 |
|  |  |  | TCGG | 0.15 | 0.08 | **0.002** |
|  |  |  | CTAG | 0.02 | 0.01 | 0.37 |
|  |  |  | TTAG | 0.03 | 0.05 | 0.07 |
|  |  |  | CCAG | 0.10 | 0.09 | 0.60 |
|  |  |  | TCAG | 0.20 | 0.20 | 0.98 |

^a^ Fisher exact test, Bonferroni correction, significant if α≤0.005. Italic font means 0.006≤P≤0.009.


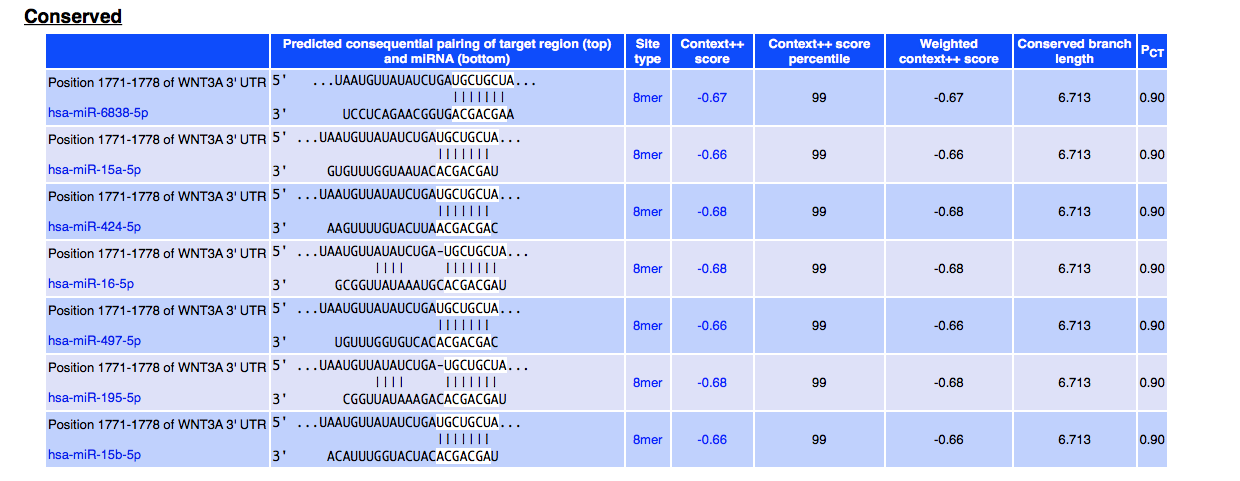


Supplementary Figure S1. Conserved miRNA binding sites for *WNT3A* according to TargetScan. Context ++ score considers the following features: site type, 3’-UTR target-site abundance, predicted seed-pairing stability, sRNA position 1, sRNA position 8, site position 8, local AU content, 3’ supplentary pairing, predicted structural accessibility, minimal distance, probability of conserved targeting, ORF length, 3’-UTR length, 3’-UTR offset-6mer sites and ORF 8mer sites. P_ct_ – probability of conserved targeting (Asgarwal *et al.*, eLife 2015).

**
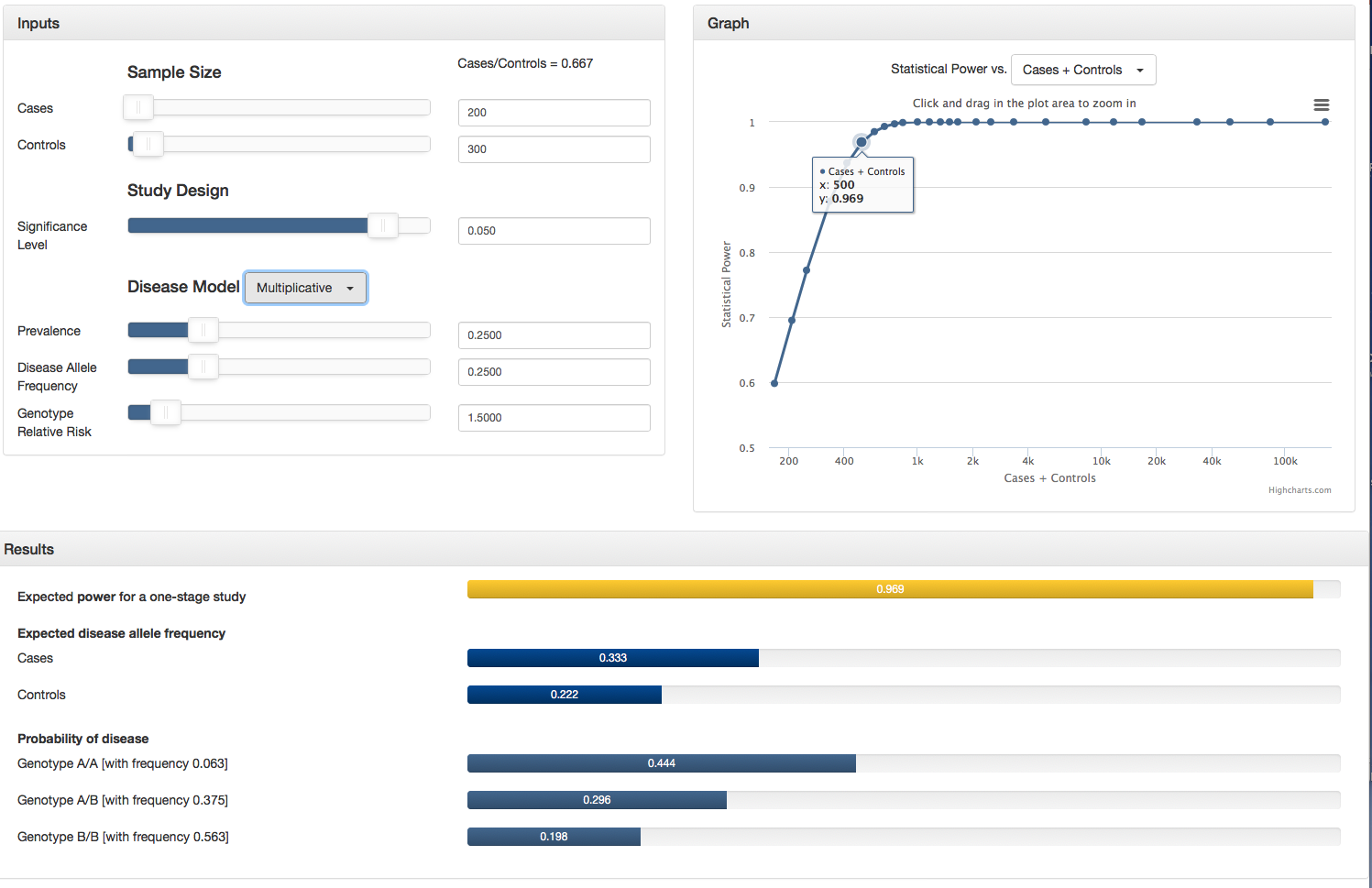
**

Supplementary Figure S2. Results of study power analysis performed with GAS Power Calculator. The following parameters were used: 1) significance level set to 0.05; 2) disease model set to multiplicative; 3) prevalence set to 0.25; 4) disease allele frequency set to 0.25; 5) genotype relative risk set to 1.5. The proposed study sample would achieve ~97% power with the used parameters.
